# CD73 alleviates GSDMD‐mediated microglia pyroptosis in spinal cord injury through PI3K/AKT/Foxo1 signaling

**DOI:** 10.1002/ctm2.269

**Published:** 2020-12-31

**Authors:** Shun Xu, Jin Wang, Junjie Zhong, Minghao Shao, Jianyuan Jiang, Jian Song, Wei Zhu, Fan Zhang, Haocheng Xu, Guangyu Xu, Yuxuan Zhang, Xiaosheng Ma, Feizhou Lyu

**Affiliations:** ^1^ Department of Orthopedics Shanghai Fifth People's Hospital Fudan University Shanghai China; ^2^ Department of Orthopedics Huashan Hospital Fudan University Shanghai China; ^3^ Department of Neurosurgery Huashan Hospital Shanghai Medical College Fudan University Shanghai China; ^4^ Neurosurgical Institute of Fudan University Fudan University Shanghai China; ^5^ Shanghai Clinical Medical Center of Neurosurgery Shanghai China; ^6^ Shanghai Key Laboratory of Brain Function and Restoration and Neural Regeneration Shanghai China

## Abstract

**Background:**

Neuroinflammation‐induced secondary injury is an important cause of sustained progression of spinal cord injury. Inflammatory programmed cell death pyroptosis executed by the pore‐forming protein gasdermin D (GSDMD) is an essential step of neuroinflammation. However, it is unclear whether CD73, a widely accepted immunosuppressive molecule, can inhibit pyroptosis via mediating GSDMD.

**Methods:**

C57BL/6J CD73 deficient mice and wild‐type mice, Lipopolysaccharide (LPS)‐induced primary microglia and BV2 cells were respectively used to illustrate the effect of CD73 on microglia pyroptosis in vivo and in vitro. A combination of molecular and histological methods was performed to assess pyroptosis and explore the mechanism both in vivo and in vitro.

**Results:**

We have shown molecular evidence for CD73 suppresses the activation of NLRP3 inflammasome complexes to reduce the maturation of GSDMD, leading to decreased pyroptosis in microglia. Further analysis reveals that adenosine‐A_2B_ adenosine receptor‐PI3K‐AKT‐Foxo1 cascade is a possible mechanism of CD73 regulation. Importantly, we determine that CD73 inhibits the expression of GSDMD at the transcriptional level through Foxo1. What's more, we confirm the accumulation of HIF‐1α promotes the overexpression of CD73 after spinal cord injury (SCI), and the increased CD73 in turn upregulates the expression of HIF‐1α, eventually forming a positive feedback regulatory loop.

**Conclusion:**

Our data reveal a novel function of CD73 on microglia pyroptosis, suggesting a unique therapeutic opportunity for mitigating the disease process in SCI.

## Graphical abstract

Inflammatory programmed cell death pyroptosis executed by the pore‐forming protein gasdermin D (GSDMD) is an essential step of neuroinflammation after spinal cord injury. We demonstrated that CD73, a widely accepted immunosuppressive molecule, can inhibit pyroptosis via mediating GSDMD through PI3K/AKT/Foxo1 signaling.

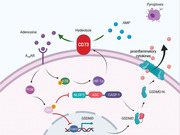

## 1 BACKGROUND

Spinal cord injury (SCI) remains a devastating condition affecting millions of people worldwide, leading to severe dysfunction below injured segment.^1^ About 240 000‐337 000 people live with SCI in United States, and this figure is thought to grow by 17 000 annually.^1^ Nearly 52% of these patients are paraplegic, and 47% are considered quadriplegic.^2^ Presently, no effective pharmacological therapy exists. The pathogenesis of SCI is complicated and can be categorized into two phases.^3^ After a transient initial mechanically inflicted trauma, a long‐lasting second phase injury happens characterized in part by secretion of cytokines and chemokines produced at the lesion site, contributing largely to neurological damage.^4^ Existing evidence demonstrates neuroinflammation exerts a key role in the secondary phase of SCI.^5^, ^6^, ^7^

Traumatic injury to central nervous system (CNS) leads to the disruption of blood spinal barrier, triggering the invasion of cells and other components of immune system cause axonal destruction, neuronal loss, and demyelination. Previous studies suggest activation of cytoplasmic inflammasome complex is essential for neuroinflammation post CNS trauma.^8^, ^9^ Inflammasomes are cytosolic multiprotein scaffolds assembled by particular pattern recognition receptors (PRRs), sensors of pathogen‐associated molecular patterns (PAMPs), and damage‐AMPs (DAMPs), enable the activation of pro‐inflammatory caspase.^10^ Presently, a number of inflammasome‐associated sensors have been discovered, such as NLRP1, NLRP3, AIM2, and pyrin.^11^ Assembly of sensor proteins, scaffolding protein apoptosis‐associated speck‐like protein containing CARD (ASC), and pro‐inflammatory caspase (caspase‐1 and ‐4/5 in humans and caspase‐1 and ‐11 in mice) into inflammasome promotes autoactivation of caspase and subsequent proteolytic gasdermin D (GSDMD) cleavage, resulting in cell pyroptosis.^10^

Pyroptosis is a proinflammatory form of programmed cell death that relies on the activity of cytosolic GSDMD driven by inflammasomes.^12^ Upon activation, GSDMD transfers to the plasma membrane and binds to the inner membrane lipids, oligomerizing to form membrane pores, resulting in local cell swelling, membrane rupture, and extravasation of cytoplasmic DAMPs.^13^, ^14^, ^15^ Released DAMPs will further recruit immune cells and aggravate inflammatory cascade.^16^ Microglia are vital mediators of innate immune responses following CNS injury.^17^ These cells are also considered to be the main cells in CNS where pyroptosis occurs.^10^, ^18^ Pyroptosis of microglia has been implicated in the pathogenesis of multiple CNS disease, including SCI and traumatic brain injury (TBI).^19^, ^20^ Although pyroptosis of microglia is repeatedly mentioned in a variety of neuroinflammatory‐related diseases, the mechanism of its occurrence is not well understood, especially in SCI.

CD73, also known as ecto‐5′‐nucleotidase (NT5E), is an AMP hydrolase that is a part of the extracellular enzymatic mechanism that regulates the conversion of extracellular ATP to adenosine.^21^ Plenty of studies have pointed to CD73 as a key regulator in various pathophysiological processes, including maintaining immune homeostasis by regulating the balance between pro‐inflammatory ATP and immunosuppressive adenosine to prevent excessive immune responses.^22^, ^23^ In addition, our previous research determined CD73 has an anti‐neuroinflammatory role in SCI, which is attributable to its regulation of macrophages/microglia polarization via adenosine‐p38 cascade.^24^ More importantly, an increasing piece of evidence now clearly indicates CD73 can suppress the activation of inflammasome.^25^, ^26^ Therefore, it is promising to clarify whether the immunosuppressive mechanism of CD73 is involved in the regulation of microglia pyroptosis after SCI.

We hypothesized that CD73 could attenuate inflammasome activation and inhibit pyroptosis of microglia, subsequently, reducing neuroinflammation after SCI. In the current study, we first confirmed the role of CD73 and GSDMD in SCI through SCI patient blood samples. Then we explored the mechanism of CD73 regulating microglia pyroptosis by in vivo and in vitro experiments. Finally, we investigated the upstream factor regulating the expression of CD73 after SCI.

## 2 METHODS

### 2.1 Donor recruitment and blood sample preparation

From January 2019 to June 2019, 20 SCI patients from Huashan Hospital, Fudan University were enrolled. Inclusion criteria included the following: (a) a clear history of trauma, and there were no neurological abnormalities of SCI before injury; (b) existing neurological abnormalities such as limb sensation, motor abnormality, and dysfunction of the bowel and bladder; (c) MRI examination showed spinal cord compression and spinal cord signal changes. Patients with treatment of methylprednisolone before blood taking, with a history of brain disease, with a history of spinal surgery were excluded. Peripheral blood was collected from 20 patients with SCI and 20 healthy donors with similar gender and age distribution, respectively. Clinical characteristics of SCI patient and healthy donors have been showed in Table 1. The Japanese orthopedic association (JOA) score, cervical dysfunction index (neck disability index [NDI]), and American spinal injury association (ASIA) classification were used to assess the severity of SCI. The whole blood RNA was extracted using GeneJET Stabilized and Fresh Whole Blood RNA Kit (ThermoFisher, CA) in accordance with the manufacturer's protocol.

**TABLE 1:** Clinical characteristics of SCI patient samples and normal control

	Total	With SCI	Without SCI	*P* value
**Patients (n)**	40	20	20	
**Ages (years)**	49.4 ± 10.4	49.7 ± 10.4	49.2 ± 10.8	.87
**Weight (kg)**	67.2 ± 7.8	66.9 ± 7.6	67.6 ± 8.1	.77
**Sex (male = 1)**				
Male	25	14	11	.51
Female	15	6	9	

### 2.2 Quantitative real‐time PCR analysis

Total RNA was extracted using TRIzol reagent (Invitrogen, San Diego, CA) according to the manufacturer's instructions, and quantification of mRNAs was performed using a 10‐μL final reaction volume using SYBR Green polymerase chain reaction (PCR) Master Mix (ThermoFisher, CA). GAPDH mRNA was used as a housekeeping gene, and relative expression levels of mRNAs were calculated using the comparative ΔΔCT method. Raw data have been supplied as File S1.

### 2.3 Animals

C57BL/6J CD73 knock out (KO) male mice used in our study were reproduced from those gifted by Prof. Thompson, Oklahoma Medical Research Foundation, Oklahoma City. Wild‐type (WT) mice used in the experiments were the descendants of the first generation which were bought from Shanghai SLAC Laboratory Animal Co., Ltd. (Shanghai, China). The specific pathogen‐free facility with a 12 hours light/dark cycle and a controlled temperature and humidity were used to house mice. All the experiments were conducted according to the protocols approved by the Institutional Animal Care and Use Committee of Fudan University.

### 2.4 Establishment of SCI model and drugs treatment

Just as descripted in our previous study,^24^ mice were anesthetized with pentobarbital by intraperitoneal injection (35 mg/kg). Each mouse was inflicted with spinal crush injury at the T8‐T9 with Dumont‐type forceps with a 0.2 mm spacer. We carried out the vertebrae laminectomies of T8‐T9 with a pair of fine rongeurs, and the dura mater of mice was protected. The mice SCI model was made by lateral compression of the spinal cord with a depth of 0.2 mm for 20 seconds. After operation, the mice were given intramuscular injection of penicillin, 20 000 units once to resist infection. For further studies the effect of AKT and HIF‐1α on SCI, SC79 (40 mg/kg in DMSO) and BAY87‐2243 (0.5 mg/kg and 4 mg/kg in DMSO) were injected intraperitoneally into mice every day after the establishment of mice SCI model. The mice in the control group were injected with the same volume of DMSO at the same time point.

### 2.5 Locomotion recover assessment

The locomotor behavior after SCI was detected by the Basso Mouse Scale (BMS) locomotor recovery scale. This scale is based on the observation of ankle movement, plantar placement, weight support, stepping, coordination, paw position, and trunk stability in an open field and consists of a 9‐point scale. Sham group, SCI+DMSO, and SCI+SC79 group were assessed on postoperative days 1, 3, 7, 14, 21, and 28. An inspector blinded to the treatments assessed them for 4 minutes in an open field and got the score from 0 (no spontaneous locomotor activity) to 9 (normal coordinated gait with parallel paw placement).

### 2.6 Cell cultures

Mixed glial cell cultures were generated from the cerebral cortex of postnatal (24 hours old) mice and cultured in Dulbecco's modified Eagle's medium/F12 containing 10% fetal bovine serum (FBS; Gibco, Carlsbad, CA) and an antibiotic mixture (1% penicillin/streptomycin) (Invitrogen, Carlsbad, CA) at 37°C and 5% CO_2_ for 10 days. Cultures were shaken for 6 hours at 180 rpm at 37°C to collect and purify microglia. BV2 cells were cultured in DMEM (Gibco, Carlsbad, CA) supplemented with 10% FBS (Gibco, Carlsbad, CA), 50 g/mL streptomycin (Invitrogen, Carlsbad, CA), and 50 U/mL penicillin in a humidified atmosphere of 95% air and 5% CO_2_. The plasmid of RNAi‐CD73, pcDNA‐CD73, and RNAi‐HIF‐1 were constructed by Genechem Co., Ltd. (Shanghai, China) and then transfected into BV2 cells using Lipofectamine 2000 reagent (Invitrogen, Carlsbad CA) following the protocol stipulated by the manufacturer. Different combinations of Lipopolysaccharide (LPS) (1 μg/mL), adenosine (10μM), MRS1706 (0.3μM), MK2206 (3μM) were added to the media 24 hours after plasmid transfection, and 8 hours later, mRNA and protein were harvested.

### 2.7 RNA sequencing and functional enrichment analysis

Total RNA was isolated from cells using the Trizol (Invitrogen, Carlsbad, CA) according to the manufacturer's protocol. RNA integrity was evaluated using the Agilent 2200 TapeStation (Agilent Technologies, Santa Clara, CA), and each sample had the RIN above 7.0. Subsequently, the purified RNAs were subjected to first strand and second strand cDNA synthesis following by adaptor ligation and enrichment with a low‐cycle according to instructions of NEBNext Ultra RNA Library Prep Kit for Illumina (NEB, Ipswich, MA). The purified l library products were evaluated using the Agilent 2200 TapeStation and Qubit2.0 (Life Technologies, Carlsbad, CA) and then diluted to 10 pM for cluster generation in situ on the pair‐end flow cell followed by sequencing (2 × 150 bp) HiSeq3000. The clean reads were obtained after removal of reads containing adapter, ploy‐N and at low quality from raw data. HISAT2 was used to align the clean reads to the mouse reference genome mm 10 with default parameters. HTSeq was subsequently employed to convert aligned short reads into read counts for each gene model. Differential expression was assessed by DEseq using read counts as input. The Benjamini‐Hochberg multiple test correction method was enabled. Differentially expressed genes were chosen according to the criteria of fold change >2 and adjusted *P*‐value <.05. All the differentially expressed genes were used for heat map analysis and Kyoto Encyclopedia of Genes and Genomes (KEGG) ontology enrichment analyses. For KEGG enrichment analysis, a *P*‐value <.05 was used as the threshold to determine significant enrichment of the gene sets.

### 2.8 Enzyme‐linked immunosorbent assay

In cell culture supernatants or mouse spinal cord homogenate, the measurement of protein levels of IL‐1β, IL‐6, and TNF‐α was taken using the commercial ELISA kits from Sigma (Sigma‐Aldrich, St. Louis, MO) according to the manufacturer's instructions.

### 2.9 Cytotoxicity assay

The release of lactate dehydrogenase (LDH) was detected to determine the cytotoxicity using the LDH Cytotoxicity Assay Kit (Beyotime, Shanghai, China) following the manufacturer's instructions.

### 2.10 Western blot analysis

Protein of spinal cord tissue and BV2 cells was homogenized in radioimmunoprecipitation assay lysis buffer, and protein concentrations were determined using the bicinchoninic acid assay. Protein samples were fractionated using sodium dodecyl sulfate (SDS)‐polyacrylamide gel electrophoresis, and transferred to nitrocellulose membranes. Following blocking for 1 hour with 5% skimmed milk in Tris Buffered saline Tween (TBST), membranes were incubated with antibodies included NLRP3 (1:1000, Abcam, ab214185), PI3K (1:1000, CST, 4228), AKT (1:1000, CST, 2920), p‐AKT (1:1000, CST, 4070), mTOR (1:1000, CST 2972S), p‐mTOR (1:1000, CST, 5536S), IKK‐β (1:1000, CST, 2678S), p‐IKK‐β (1:1000, CST, 2697S), GSK‐3β (1:1000, CST, 2456S) p‐GSK‐3β (1:1000, CST, 5558S), Bad (1:1000, CST, 9268S), p‐Bad (1:1000, CST, 5284S), Foxo1 (1:1000, CST, 14952), p‐Foxo1 (1:1000, CST, 9641), GSDMD (1:1000, Abcam, ab210070), ASC (1:1000, CST, 67824), and GAPDH (1:2000, Abcam, ab8245) overnight at 4°C. After being washed in TBST, membranes were incubated with HRP‐conjugated secondary antibody at room temperature for 1 hour, and protein was detected using an enhanced chemiluminescence kit. Signal intensities were quantified by gel imaging system (UVP LLC, Upland, CA). The quantification of blots was performed by Image J.

### 2.11 Immunohistochemical assessment

At the third day after SCI, different groups' mice were deeply anesthetized with 10 % chloralic hydras (3.5 mL/kg, i.p.) and perfused with 0.9 % NaCl, followed by 4 % paraformaldehyde in 0.01 M phosphate‐buffered saline (PBS, pH = 7.4). Spinal cord segments near the lesion epicenter were collected. Three transverse paraffin sections (25μm thick) were mounted on poly‐L‐lysinecoated slides. For immunohistochemical analysis, deparaffinized sections were incubated with H_2_O_2_ and methanol for 10 minutes to block endogenous peroxidase, and then incubated for 30 minutes with serum‐blocking solution. The sections were then incubated with primary antibodies of CD73 (1:100, Abcam, ab175396), GSDMD (1:100, Abcam, ab210070), and CASP‐1 (1:100, Abcam, ab1872) for 1 hour, followed by incubation with HRP‐conjugated anti‐rabbit secondary antibodies for 30 minutes. Bound antibodies were visualized by incubation with 3,3N‐Diaminobenzidine Tertrahydrochlori for 10 minutes. All images were captured using a Nikon ECLIPSE Ti microscope (Nikon, Japan).

### 2.12 Immunofluorescence assessment

Spinal cord tissue samples were harvested as described above. BV2 cell samples were fixed with 4% paraformaldehyde in 0.1 M phosphate buffer (pH 7.4) for 15 minutes after the treatment described in cell cultures. Then all samples were blocked for 1 hour with 1 % bovine serum albumin containing 0.3 % Triton X‐100, followed by incubation (overnight at 4°C) with GSDMD (1:100, Abcam, ab210070) and CASP‐1 (1:100, Abcam, ab1872) the primary antibodies. Then all the samples were incubated (for 2 hours at room temperature) with their respective secondary antibody: Dylight (Dy)488‐ and Dy594‐conjugated secondary antibodies (all 1:1000; Jackson ImmunoResearch, West Grove, PA). All images were acquired using Nikon ECLIPSE Ti microscope (Nikon, Japan).

### 2.13 Promoter cloning and generation of dual‐luciferase reporter assay

A −2000/+200 bp mouse GSDMD promoter of C57BL/6J mouse genomic DNA was cloned and inserted in the pGL3 basic vector (Promega, Madison, WI). Further deletion generated −1450/+200 bp, −900/+200 bp, −300/+200 bp, +50/+200 bp reporter plasmids. Mutant GSDMD reporter plasmids were generated based on −300/+200 bp plasmid. BV2 cells were cotransfected with luciferase reporter plasmids, pRL‐TK reporter plasmid (control reporter), and foxo1 plasmid (pc‐foxo1). After transfection for 24 hours, cells were harvested and measured using the toolVeritas 9100‐002 (Turner BioSystems, Sunnyvale,CA), and luciferase activity was divided by the Renilla luciferase activity to normalize for transfection efficiency.

### 2.14 Chromatin immunoprecipitation assay

ChIP assay was performed using a ChIP assay kit (Abcam, Cambridge, UK) according to the manufacturer's protocol. Per condition, 10^7^ cells were treated with 1% formaldehyde for 10 minutes prior to three washes with ice‐cold PBS. Cells were then resuspended in 5 mL SDS lysis buffer containing 1 × protease inhibitor cocktail II and sonicated 15 times with 20 seconds pulses at 4°C. Insoluble material was removed by centrifugation for 10 minutes at 12 000 xg, 4°C. For each immunoprecipitation (IP), 100 μL of prepared chromatin was added to 900 μL of dilution buffer. The lysates were then pre‐cleared with Protein G agarose before being incubated with appropriate antibodies overnight at 4°C. The following day 60 μL of Protein G agarose was added to each IP and rotated for 1 hour at 4°C. The Protein G agarose‐antibody/chromatin complexes were then washed sequentially in 1 mL of the provided buffers. The DNA/protein complexes were eluted in 200 μL elution buffer before reversal of crosslinks with 5 M NaCl for 4 hours at 65°C. Samples were then treated with RNase A at 37°C for 30 minutes followed by incubation with Proteinase K for 2 hours at 45°C. The resulting DNA was purified over the provided spin filter columns, with elution in 50 μL of elution buffer C. The purified DNA was then subjected to quantitative Polymerase Chain Reaction (qPCR). Each qPCR reaction performed in triplicate containing 5 μL purified DNA and was quantified as described above.

### 2.15 Statistical analysis

All results were expressed as mean ± standard deviation. Student's unpaired *t*‐tests and two‐way analysis of variance (ANOVA) followed by Dunnett's test were used to analyze data. A *P* value of less than .05 was considered to be statistically significant. All statistical analyses were done with the SPSS 14.0 software.

## 3 RESULTS

### 3.1 Expression of NLRP3/GSDMD genes in peripheral blood of patients with SCI is positive correlated with the severity of injury

**FIGURE 1:**
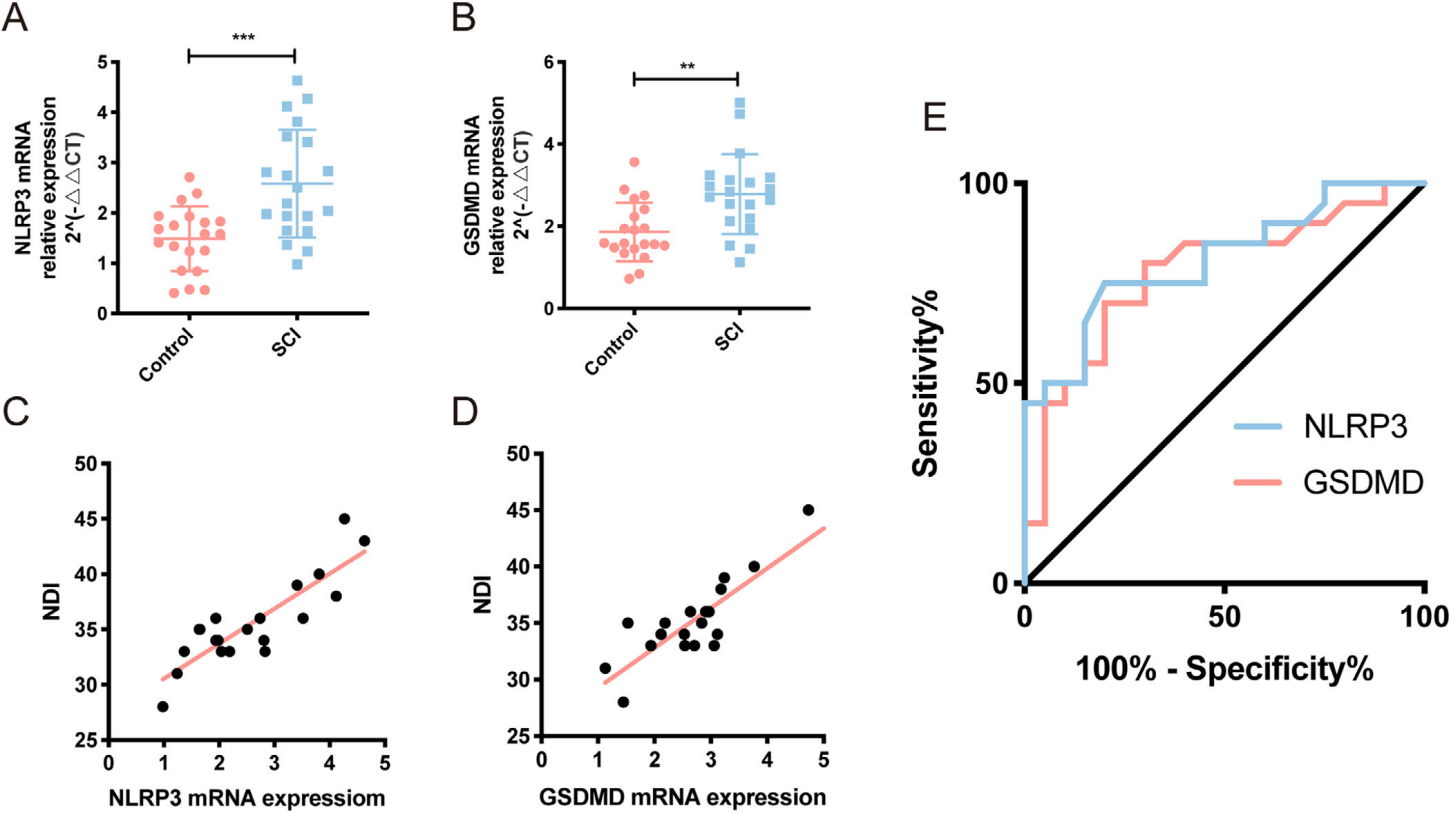
Expression of NLRP3 and GSDMD genes in peripheral blood of patients with SCI is positive correlated with the severity of injury. **A and B,** Relative mRNA expression of NLRP3 and GSDMD was assessed by RT‐PCR in 20 normal people and 20 SCI patients. **C,** The linear regression analysis of the relevance between NLRP3 mRNA expression and JOA score (r = −0.81, *P* < .001), and NDI index (r = 0.86, *P* < .001). **D,** The linear regression analysis of the relevance between GSDMD mRNA expression and JOA score (r = −0.88, *P* < .001), and NDI index (r = 0.87, *P* < .001). **E,** The ROC curve analysis the diagnostic significance of NLRP3 (AUC = 0.74) and GSDMD (AUC = 0.72) for SCI

**FIGURE 2:**
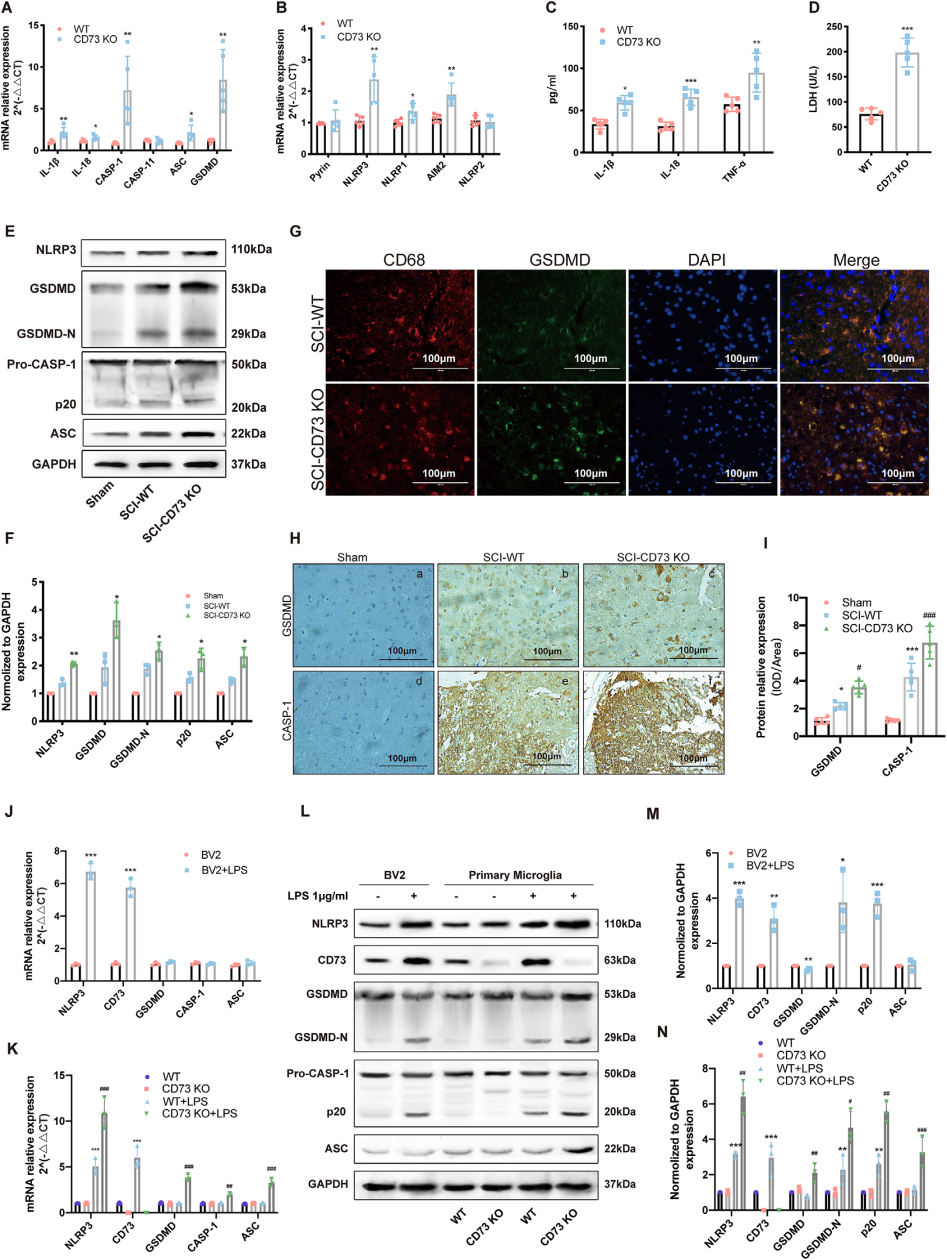
CD73 deficiency facilitates NLRP3 inflammasome activation and pyroptosis of microglia in vivo. **A and B,** Relative mRNA expression of pyroptosis‐related genes on the third day after SCI in WT and CD73 KO mice (N = 5). **C,** The release of IL‐1β, IL‐6, and TNF‐α in spinal cord homogenate was measured by ELISA. **D,** The release of LDH was detected by LDH Assay Kit (N = 5). **E and F,** Representative western blotting and statistical comparison of pyroptosis‐related protein on the third day after SCI or sham surgery in WT or CD73‐deficient mice (N = 3). **G,** Representative two‐photon excitation images of immunofluorescence of CD68 and GSDMD acquired from WT or CD73 KO mice 3 days post‐injury or sham surgery. **H and I,** Immunopositive particles for GSDMD and CASP‐1 and quantification 3 days post‐injury or sham surgery in WT or CD73 KO mice (N = 5). **J and K,** Relative mRNA expression of pyroptosis‐related genes in BV2 cells or primary microglia with or without LPS (1μg/mL, 8 hours) treatment (N = 3). **L‐N,** Representative western blotting and statistical comparison of pyroptosis‐related protein in BV2 cells and primary microglia with or without LPS (1μg/mL, 8 hours) treatment (N = 3). *P < .05, **P < .01, ***P < .001. All data are shown as the mean ± SD independent experiments

To investigate whether NLRP3/GSDMD plays a role in neuroinflammation after SCI, 40 blood samples (20 normal vs 20 SCI) were subjected to RT‐PCR assay. Then 20 SCI patients were divided into high‐expression and low‐expression groups based on whether NLRP3/GSDMD gene expression reached an average level. As shown in Table 2, various clinical parameters in SCI patients were compared between two groups. Specifically, NLRP3/GSDMD high‐expression group had a lower JOA score and a higher NDI compared to NLRP3/GSDMD low‐expression group. Strikingly, quantitative analysis revealed that the expression of NLRP3/GSDMD was significantly higher in the SCI patient samples in comparison to normal samples (Figures 1A and 1B). Furthermore, linear regression analysis confirmed a positive correlation between NLRP3/GSDMD expression and JOA score, while a negative correlation between CD73/GSDMD expression and NDI index (Figures 1C and 2D). The receiver operating characteristic curve (ROC) curve further demonstrated they may be good diagnostic markers for SCI clinically (Figure 1E). Collectively, all these above results indicate the expression level of CD73 and GSDMD in SCI patients correlate with the severity of injury.

**TABLE 2:** Clinical characteristics of patient samples and expression of NLRP3 and GSDMD

		NLRP3			GSDMD		
	Total	High	Low	*P* value	High	Low	*P* value
**Patients (n)**	20	9	11	–	10	10	–
**Sex (n)**	20	9	11	.49	10	10	1
**Age (years)**	49.7 ± 10.3	51.3 ± 12.1	48.4 ± 9.1	.54	51.9 ± 11.5	57.5 ± 9.1	.36
**Weight (kg)**	66.9 ± 7.6	69.2 ± 5.8	64.9 ± 8.6	.22	67.4 ± 7.9	66.3 ± 7.7	.76
**NDI**	35.6 ± 3.9	38.2 ± 3.9	33.6 ± 2.2	.03	37.9 ± 3.9	33.2 ± 5.3	.04
**ASIA (n)**							
a	1	1	0	.105	1	0	.306
b	7	5	2		5	2	
c	9	3	6		3	6	
d	3	0	3		1	2	
e	0	0	0		0	0	

### 3.2 CD73 deficiency facilitates NLRP3 inflammasome activation and pyroptosis of microglia in vivo

To examine the inflammasome expression in SCI systematically, the inflammasome‐related genes were assessed in spinal cord tissue samples from mice with SCI or sham surgery. All inflammasome‐associated genes were detectable in mice spinal cord tissue 3 days after surgery, with increased IL‐1β, IL‐18, CASP‐1 and GSDMD transcript levels in SCI group (Figure S1A). Besides, all inflammasome sensors examined were also detectable, and NLRP3 and AIM2 transcript levels were upregulated (Figure S1B) after SCI. To investigate the influence of CD73 on inflammasome activation, the mRNA detection of the above genes was performed once again in CD73 KO mice with SCI. As shown in Figures 2A and 2B, compared with WT mice, various inflammasome‐associated genes (IL‐1β, IL‐18, CASP‐1, ASC, and GSDMD) as well as three inflammasome sensors (NLRP3, NLRP1, and AIM2) showed higher levels in CD73 KO mice 3 days post‐injury. In addition, the release activity of proinflammatory factor (IL‐1β, IL‐6, and TNF‐α) and LDH were also effectively increased in CD73 KO mice after SCI (Figures 2C and 2D). Consistently, western bolt and immunochemistry revealed similar protein expression patterns of NLRP3, GSDMD, ASC, and CASP‐1 as their mRNA expression (Figures 2E, 2F, and 2H). In particular, levels of full length GSDMD, N‐terminal GSDMD (GSDMD‐N, an active form of GSDMD), p20 (an active form of CASP‐1), were increased (Figures 2E and 2F). More importantly, Immunofluorescence results (Figure 2G) showed that the increased expression of GSDMD coincides with CD68 (a marker of microglia) suggesting the cell pyroptosis was occurred mainly in microglia. In addition, the inflammasome‐related genes were assessed in BV2 cells and primary microglia. As shown in Figures 2J, 2L, and 2M, the treatment with LPS increased NLRP3 and CD73 gene levels, facilitated the activation of GSDMD and CASP‐1. Consistently, the treatment of LPS had similar effect on primary microglia (Figures 2K, 2L, and 2N). Particularly, NLRP3, GSDMD, CASP‐1, and ASC showed higher levels in primary microglia from CD73 KO mice (Figures 2K, 2L, and 2N). Together, these results demonstrate that CD73 deficiency exacerbates microglia pyroptosis, and this effect may relate to NLRP3 inflammasome.

### 3.3 CD73 alleviates LPS‐induced NLRP3 inflammasome activation and pyroptosis in BV2 cells through A2B adenosine receptor

**FIGURE 3:**
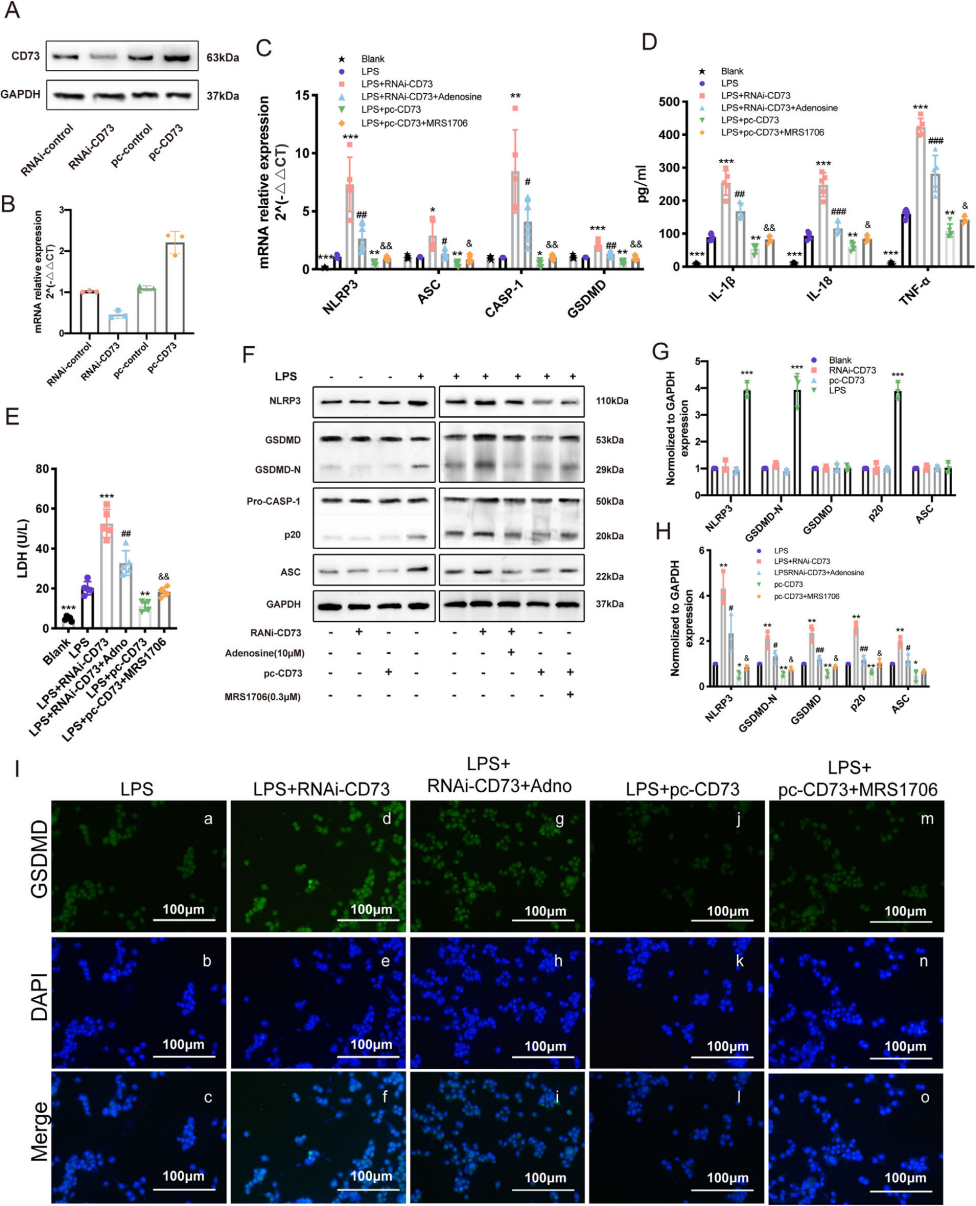
CD73 alleviates LPS‐induced NLRP3 inflammasome activation and pyroptosis in BV2 cells through A_2B_AR. After small interference RNA (RNAi) or −CD73 transfection for 24 hours, BV2 cells were challenged with LPS (1μg/mL) for another 8 hours in presence of adenosine (10μM) or MRS1706 (1μM). **A,** The CD73 protein expression in small interference RNA or pc‐CD73 reansfected BV2 cells. **B,** Relative mRNA expression of CD73 in small interference RNA or pc‐CD73 transfected BV2 cells (N = 3). **C,** After administration of LPS combined with adenosine or MRS1706, microglial pyroptosis markers (NLRP3, ASC, CASP‐1, and GSDMD) mRNA expression were identified by RT‐PCR in CD73 downregulated or upregulated BV2 cells (N = 5). **D,** The release of IL‐1β, IL‐18, and TNF‐α in different cultured groups measured by ELISA (N = 5). **E,** The release of LDH was detected by LDH Assay Kit in different cultured groups (N = 5). **F‐H,** The pyroptosis‐related protein expression and statistical comparison in BV2 cells in different treated groups(N = 5). **I,** Representative images of immunofluorescence of GSDMD acquired from different treated BV2 cells. ^*^ *P* < .05, ^**^ *P* < .01, ^***^ *P* < .001 versus control; ^#^ *P* < .05, ^##^ *P* < .01, ^###^ *P* < .001 versus RNAi‐CD73; ^&^ *P* < .05, ^&&^ *P* < .01 versus pc‐CD73. All data are shown as the mean ± SD independent experiments

Our previous studies have shown that CD73 regulates microglia polarization through the A_2B_ adenosine receptor (A_2B_AR).^24^ Therefore, to determine whether A_2B_AR also mediates the CD73's ability to alleviate microglia pyroptosis, we constructed BV2 cells that either had downregulated or upregulated CD73 expression, exposed them with adenosine or A_2B_AR antagonist MRS1706 after LPS pretreatment. Immunoblot and RT‐PCR results showed that CD73 interference and overexpression had acceptable efficiency (Figures 3A and 3B). Results of RT‐PCR and ELISA showed that CD73 downregulation caused a profound induction of pyroptosis genes in transcript levels including NLRP3, ASC, CASP‐1, and GSDMD, increased the release of IL‐1β, IL‐18, TNF‐α, and LDH, while CD73 upregulation was counterproductive (Figures 3C and 3E). Immunoblot and Immunofluorescence revealed similar changes in protein levels of NLRP3, GSDMD, and ASC replying to CD73 change (Figures 3F and 3I). To further determine the role of A_2B_AR in the CD73 mediated BV2 cell pyroptosis, the cells were exposed with adenosine or MRS1706 (A_2B_AR antagonist) after LPS pretreatment. Consequently, the presence of adenosine further alleviates the pyroptosis induced by CD73 upregulation, while the addition of MRS1706 obviously abolishes the effects of CD73 upregulation in all above experiments, respectively (Figures 3C and 3I). All these results suggest that A_2B_AR may necessary for CD73 to regulate microglia pyroptosis.

### 3.4 CD73 attenuates microglia pyroptosis via PI3K/AKT/Foxo1 signal

**FIGURE 4:**
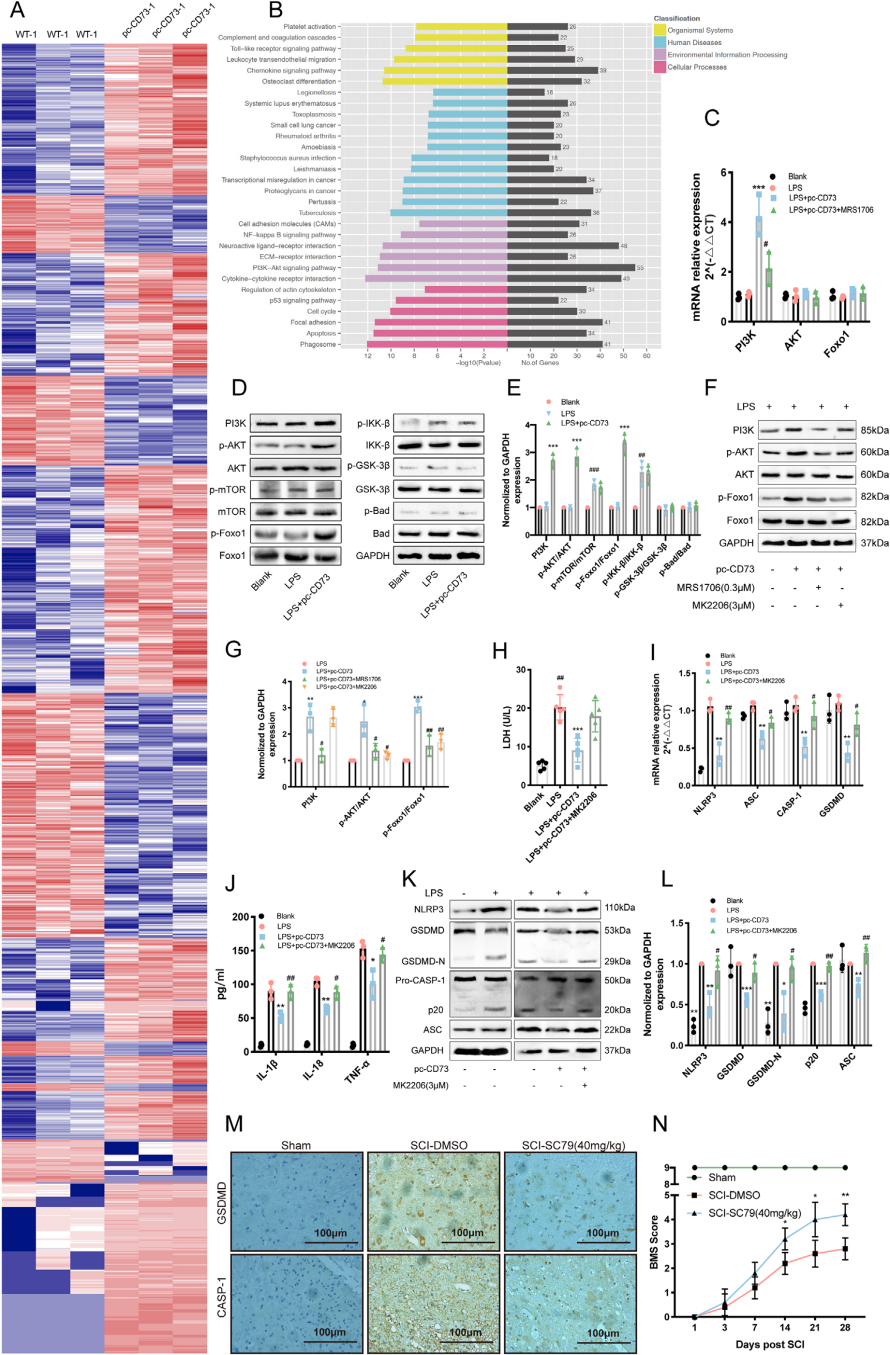
CD73 attenuates microglia pyroptosis via PI3K/AKT/Foxo1 signal. After overexpression plasmid transfection for 24 hours, BV2 cells were challenged with LPS (1μg/mL) for another 8 hours, then 3 independent samples, and their controls were RNA sequenced. **A,** The heat map analysis of differentially expressed mRNAs. **B,** The KEGG ontology enrichment analyses of differentially expressed mRNAs. **C,** The mRNA expression of PI3K, AKT, Foxo1 in BV2 cells challenged with/without LPS in presence or absence of pc‐CD73 or MK2206 (3μM) (N = 3). **D and E,** The expression and phosphorylation of PI3K, AKT, mTOR, Foxo1, IKK‐β, GSK‐3β, Bad and statistical comparison in BV2 cells in different treated groups (N = 3). **F and G,** The expression and phosphorylation of PI3K, AKT, Foxo1 and statistical comparison in BV2 cells in different treated groups (N = 3). **H,** The release of LDH was detected by LDH Assay Kit in different cultured groups (N = 5). **I,** The microglial pyroptosis markers (NLRP3, ASC, CASP‐1, and GSDMD) mRNA expression was identified by RT‐PCR in CD73 upregulated BV2 cells in presence of MK2206 (3μM) or not (N = 3). **J,** The release of IL‐1β, IL‐18, and TNF‐α in different cultured groups measured by ELISA (N = 3). **K and L,** The pyroptosis‐related protein expression and statistical comparison in BV2 cells in different treated groups (N = 3). ^*^ *P* < .05, ^**^ *P* < .01, ^***^ *P* < .001 versus control; ^#^ *P* < .05, ^##^ *P* < .01, ^###^ *P* < .001 versus pc‐CD73. Intraperitoneal injection of 40 mg/kg SC79 or DMSO was carried out every day after surgery in CD73 KO mice. **M,** Representative immunohistochemical staining for GSDMD and CASP‐1 on the third day after SCI or sham surgery. **N,** Degree of motor disturbance assessed by the Basso Mouse Scale (BMS) criteria at different time points after SCI with SC79 treatment or not (N = 6). ^*^ *P* < .05, ^**^ *P* < .01. All data are shown as the mean ± SD independent experiments

To further investigate the mechanism by which CD73 regulates microglia pyroptosis, mRNA sequencing was performed on CD73‐overexpressing BV2 cells after administrated with LPS. A total of 1649 mRNAs were differentially expressed (1081 upregrated and 568 downregulated) between CD73 overexpressed BV2 cells and normal subjects (Figure 4A). Notably, 55 differentially expressed mRNAs were enriched in PI3K/AKT pathway (Figure 4B). To explore whether CD73 regulates pyroptosis through PI3K/AKT signal pathway, the expression of PI3K, AKT, and the common downstream signaling molecules of it (including mTOR, IKK‐β, GS3K‐β, Bad, and Foxo1) were detected in CD73 overexpressed BV2 cells (pretreated with LPS or not). As showed in Figures 4D and 2E, LPS treatment promoted the phosphorylation of mTOR and IKK‐β, while CD73 overexpression only increased the activation of Foxo1. Then the RT‐PCR and immunoblot were used to confirm the relationship between CD73 and PI3K/AKT/Foxo1 axis. As indicated in Figures 4C, 4F, and 4G, the mRNA and protein expressions of PI3K were all increased in CD73 overexpressed BV2 cells, and the elevated level of phosphorylated AKT and Foxo1 were also observed. In addition, we found the mRNA level of PI3K, the phosphorylated AKT and Foxo1were reversed after exposed to MRS1706 in CD73 upregulated cells, and their expression could also be suppressed by an AKT inhibitor MK2206 (Figures 4C, 4F, and 4G). Moreover, MK2206 treatment also caused a remarkably increase in the pyroptosis‐associated genes (including NLRP3, ASC, CASP‐1, and GSDMD) in transcript levels, as well as increased the release of IL‐1β, IL‐6, TNF‐α, and LDH (Figures 4H and 4J). Similar changes in protein levels of NLRP3, GSDMD, and ASC were also observed by immunoblot replying to MK2206 (Figures 4K and 4L). In vivo study, the SC79 (an AKT activator) was injected intraperitoneally (40 mg/kg) once a day after SCI in CD73 KO mice, and the results of immunohistochemistry proved that SC79 could decrease the expression of GSDMD and CASP‐1 at the lesion site (Figure 4M). Furthermore, the BMS scoring in SC79‐treated group was significantly superior to DMSO‐treated group at 28th day after injury (Figure 4N). Taken together, we conclude that CD73 could alleviate microglial pyroptosis in vitro and in vivo, and the effect may mediate via the PI3K/AKT/Foxo1 pathway.

### 3.5 Foxo1 is a transcriptional activator in the promoter region of GSDMD gene

**FIGURE 5:**
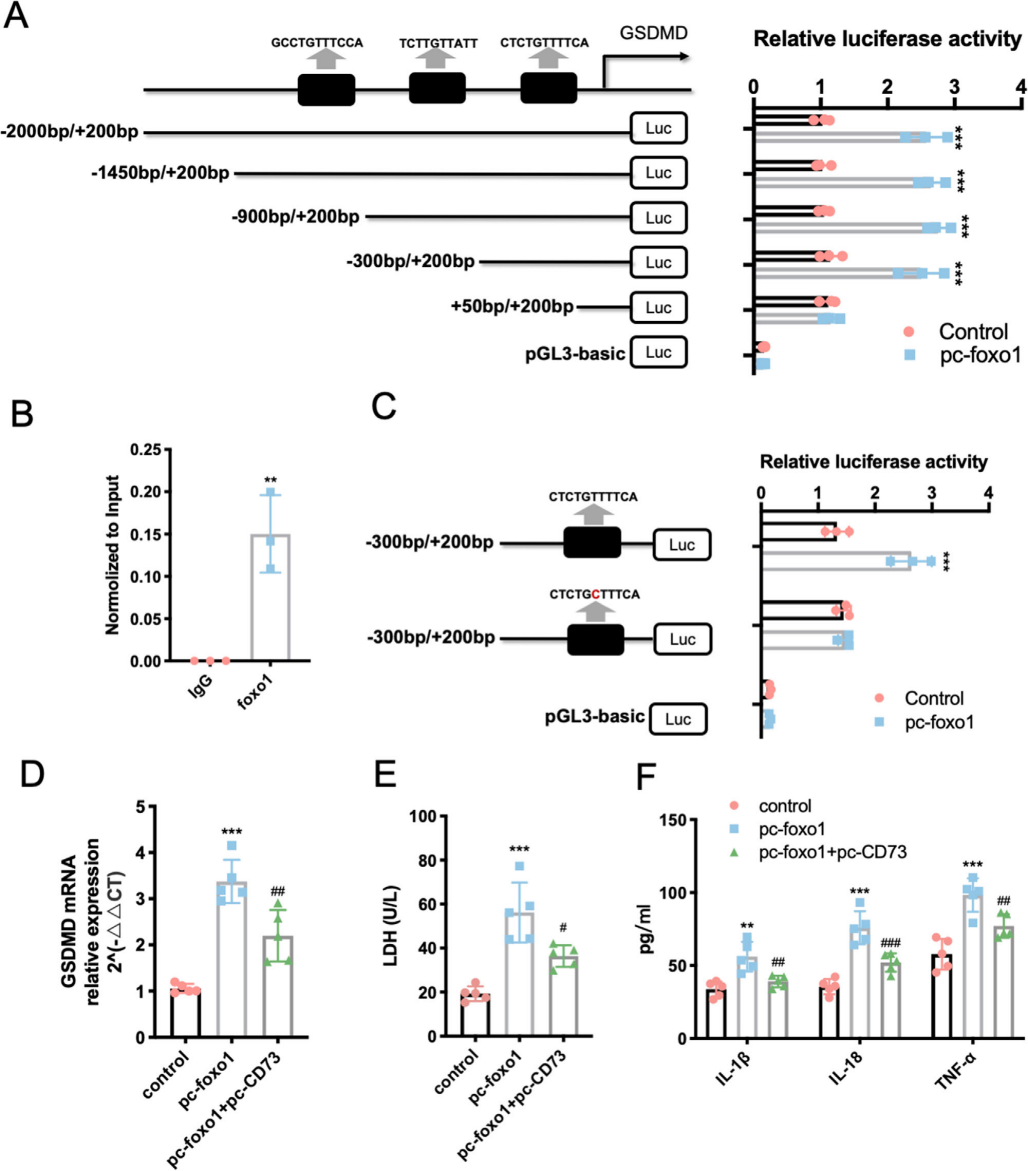
Foxo1 is a transcriptional activator in the promoter region of GSDMD gene. A series of plasmids containing 5′ unidirectional deletions of the promoter region of the GSMDM gene fused in frame to luciferase gene were transfected into BV2 cell lines as well as pc‐Foxo1. Luciferase assay was performed after 48 hours. **A,** Dual‐luciferase reporter assay of GSDMD and Foxo1 (N = 3)**. B,** Result of ChIP analysis between GSDMD and Foxo1 (N = 3). **C,** The mutant GSDMD promoter‐driven luciferase reporters with mutated bases shown in red were constructed, and relative fluorescence intensity was measured (N = 3). **D,** The mRNA level of GSDMD of BV2 cells after incubated with pc‐Foxo1 in presence with pc‐CD73 or not for 24 hours (N = 3). **E,** The release of LDH was detected by LDH Assay Kit in different cultured groups. **F,** The release of IL‐1β, IL‐6, and TNF‐α in different cultured groups measured by ELISA (N = 3). ^**^ *P* < .01, ^***^ *P* < .001 versus control; ^#^ *P* < .05, ^##^ *P* < .01, ^###^ *P* < .001 versus pc‐ Foxo1. All data are shown as the mean ± SD independent experiments

Foxo1 is a transcriptional factor and widely participates in the transctipiton process of many genes. In order to continue analyze the molecular mechanisms involved in macroglia pyroptosis, we used the JASPAR database (http://jaspar.genereg.net) to identify potential binding sites of GSDMD promoter region for Foxo1. To measure the activity of potential 3 cis‐acting elements and determine the minimum sequence required for activity, a series of five reporters constructs with progressively larger deletions from the 5′ end of the promoter were generated. Luciferase assay revealed an increased promoter activity of the pGL3–2000/+200 bp as compared to the control group in BV2 cells, indicating the existence of binding site for Foxo1 in the promoter region of GSDMD gene (Figure 5A). Consistently, the ChIP assay confirmed the direct binding of Foxo1 to GSDMD promoter region (Figure 5B). However, when the promoter sequence was deleted to position +50 bp, the promoter activity with pGL3+50/+200 bp decreased significantly compared with the pGL3−300/+200 (Figure 5A), this result demonstrated that positive regulatory elements were located in the −300/+50 promoter region of GSDMD in BV2 cells. As expected, further mutation of this binding site effectively blocked the transcription of GSDMD (Figure 5C). Overexpression of Foxo1 in BV2 cells also increased the expression of GSDMD, while cotreatment of Foxo1 plasmid and CD73 plasmid inhibited the elevation of GSDMD mRNA, as well as reduced the release of IL‐1β, IL‐6, TNF‐α, and LDH (Figures 5D and 5F). Thus, we conclude that Foxo1 mediates the pyroptosis inhibition effect of CD73 by regulating the expression of GSDMD at transcriptional level.

### 3.6 HIF‐1α mediates CD73 upregulation in microglia

**FIGURE 6:**
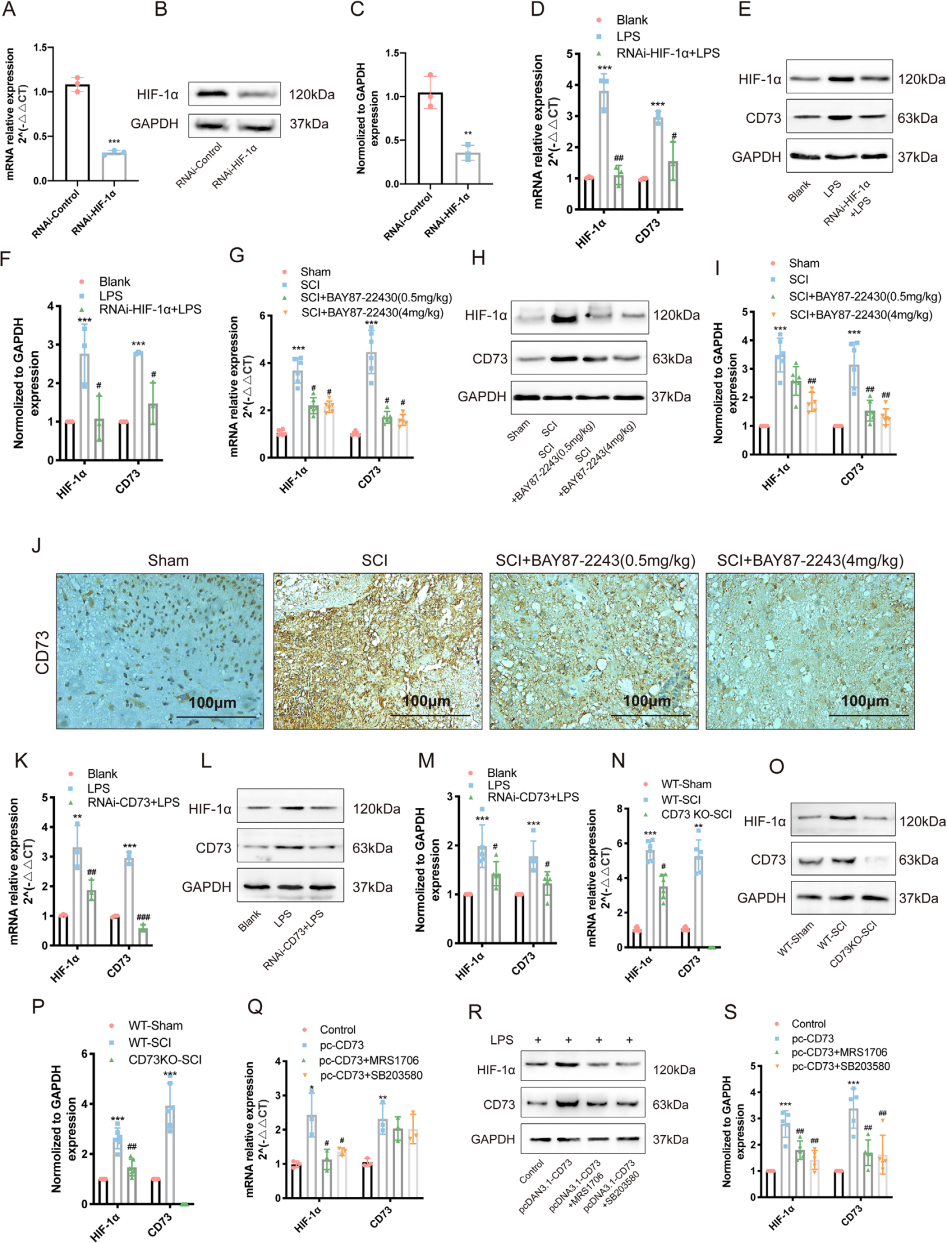
HIF‐1α mediates CD73 upregulation in microglia. **A,** Relative mRNA expression of HIF‐1α in small interference RNA transfected BV2 cells (N = 3). **B and C,** The HIF‐1α protein expression and statistical comparison in BV2 cells with or without small interference RNA transfected (N = 3). **D,** The mRNA expression of HIF‐1α and CD73 in BV2 cells challenged with LPS in presence or absence of RNAi‐HIF‐1α (N = 3). ^***^ *P* < .001 versus blank; ^#^ *P* < .05, ^##^ *P* < .01 versus LPS group. **E and F,** The HIF‐1α and CD73 protein expression in BV2 cells challenged with LPS in presence or absence of RNAi‐HIF‐1α and statistical comparison (N = 3). ^***^ *P* < .001 versus blank; ^#^ *P* < .05 versus LPS group. **G,** The mRNA expression of HIF‐1α and CD73 in WT mice 3 days post SCI, in which intraperitoneal injection of BAY87‐22430 or DMSO was carried out every day after surgery (N = 6). ^***^ *P* < .001 versus Sham; ^#^ *P* < .05 versus SCI. **H and I,** The HIF‐1α and CD73 protein expression in WT mice of different groups and statistical comparison (N = 6). ^***^ *P* < .001 versus Sham; ^##^ *P* < .01 versus SCI. **J,** Immunopositive particles for CD73 3 days post‐injury in WT mice with or without BAY87‐2243 treatment. **K,** The mRNA expression of HIF‐1α and CD73 in BV2 cells challenged with LPS in presence or absence of RNAi‐CD73 (N = 3). ^**^ *P* < .01, ^***^ *P* < .001 versus blank; ^##^ *P* < .01, ^###^ *P* < .001 versus LPS group. **L and M,** The HIF‐1α and CD73 protein expression in BV2 cells challenged with LPS in presence or absence of RNAi‐CD73 and statistical comparison (N = 3). ^***^ *P* < .001 versus blank; ^#^ *P* < .05 versus LPS group. **N,** The mRNA expression of HIF‐1α and CD73 in WT or CD73 KO mice 3 days post‐SCI (N = 6). ^**^ *P* < .01, ^***^ *P* < .001 versus WT‐Sham; ^#^ *P* < .05 versus WT‐SCI. **O and P,** The HIF‐1α and CD73 protein expression in WT or CD73 KO mice 3 days post‐SCI (N = 6). ^***^ *P* < .001 versus WT‐Sham; ^##^ *P* < .01 versus WT‐SCI. **Q,** After 24 hours pretreated with pc‐CD73, the mRNA expression of HIF‐1α and CD73 in BV2 cells with determined by RT‐PCR after challenged with LPS combined with MRS1706 (1μM) or SB203580 (10μM) for another 8 hours (N = 3). ^*^ *P* < .05, ^**^ *P* < .01 versus control; ^#^ *P* < .05 versus pc‐CD73. **R and S**. The HIF‐1α and CD73 protein expression in different treated BV2 cells (N = 5). ^***^ *P* < .001 versus control; ^##^ *P* < .01 versus pc‐CD73. All data are shown as the mean ± SD independent experiments

Several reports have shown that HIF‐1α can regulate CD73 expression.^27^ So, in this study, we exposed LPS‐activated BV2 cells with a HIF‐1α small interference RNA to determine its regulatory role. Immunoblot and RT‐PCR results showed that HIF‐1α interference had acceptable efficiency (Figures 6A and 6C). LPS‐induced pyroptosis could simultaneously promote the expression of HIF‐1α and CD73 in BV2 cells, and the overexpression of CD73 was inhibited after interference of HIF‐1α (Figures 6D and 6F). In vivo, the results also indicated that SCI could increase the expression of HIF‐1α along with the elevation of CD73, while BAY87‐2243 treatment, a HIF‐1α inhibitor, reversed the expression pattern of CD73 (Figures 6G and 6J). Surprisingly, we found that the BAY87‐2243, which was supposed to only affect the HIF‐1α activity, also reduced HIF‐1α expression both in mRNA and protein levels (Figures 6G and 6J). We suspect that CD73 reduction should be responsible for this phenomenon. As we expect, the increase of HIF‐1α induced by LPS or SCI was blocked in CD73 downregulated BV2 cells and in CD73 knockout mice, respectively (Figures 6K and 6P). Moreover, Merighi et al reported a stimulatory effect of adenosine on HIF‐1α by activation of p38 mitogen‐activated protein kinases phosphorylation via A_2B_ adenosine receptor.^28^ Our previous study also demonstrated that CD73 can regulate the polarization of macrophages/microglia through this pathway.^24^ This prompted us to verify whether CD73 regulates HIF‐1α expression is associated with this signal. As suggested in Figures 6Q and 6S, the results of RT‐PCR and immunoblots showed that both a A_2B_ adenosine receptor inhibitor MRS1706 and a p38 inhibitor SB203580 can reduce the increase in HIF‐1α caused by overexpression of CD73 in BV2 cells. In general, the above studies confirmed that HIF‐1α and CD73 can promote each other and subsequently jointly regulate microglia pyroptosis.

## 4 DISCUSSION

CNS trauma, either SCI or TBI, can be divided into two stages (primary injury and secondary injury) according to its characteristics of pathological process.^29^ Part of the secondary injury is characterized by persistence and diffuseness, including delayed glial and neuron cells death, leading to significant expansion of the damage site to higher segments and progressive neurodegeneration.^30^, ^31^ Neuroinflammation initiated by the innate immune response plays an important role in the pathology of secondary injury after CNS trauma, and inhibition of inflammation becomes a potential therapy for such injury.^26^ More recently, the scientific community has determined that the activation of cytoplasmic inflammasome complexes leading to pyroptosis was an essential step of neuroinflammation in secondary CNS damage.^7^ Although inflammasomes have been proposed in SCI, the effect of the pyroptosis‐executing protein GSDMD on SCI was not clear. In addition, whether CD73, a 5′‐ecto‐nucleotidase which can attenuate neuroinflammation after SCI determined by our previous study, can affect the expression of GSDMD has not been studied. Here, we showed that NLRP3 and GSDMD were significantly upregulated in the blood samples from SCI patients, and they were also correlated with the severity of this disease (Figures 1A and 1D). Moreover, ROC curve identified these genes have the potential to be a novel target for the diagnosis of human SCI (Figure 1E). These results confirmed our speculation about the involvement of NLRP3 and GSDMD in SCI.

In generally, inflammasome complexes consist of three parts: a cytosolic pattern‐recognition receptor, pro‐inflammatory caspase, and an adaptor protein ASC that facilitates the interaction between the two.^11^, ^32^ Among the multiple inflammasome complexes, NLRP3 appears to be one of the most relevant components in CNS trauma.^33^ Results of the research carried out by Liu et al indicated NLRP3 inflammasome was overexpressed in microglia, neurons, and astrocytes in TBI rats.^34^ The experimental results of Wu et al demonstrated that pharmacologic suppression of NLRP3 inflammasome activation controls neuroinflammation, attenuates mitochondrial dysfunction in mice.^19^ In addition, Adamczak and colleagues found AIM2 was expressed in cortical neurons and could be activated by TBI.^35^ In this study, we systematically examined the expression of inflammasome‐associated genes and found NLRP3 and AIM2 were overexpressed in spinal cord tissue after injury. What's more, the increase in NLRP3 was more pronounced, confirming that it may play a more important role after SCI, which was consistent with previous findings (Figure S1). To estimate the effects of CD73 on inflammasome pathway in SCI, we compared inflammasome‐related gene expression profile between CD73 KO mice and WT mice. We found that CD73 deficiency promoted the expression of inflammasome genes, as well as increased the release of pro‐inflammatory. What needs to be pointed out was that all the experimental animals used here were C57BL/6J strain and were housed in the same specific pathogen‐free facility to ensure the reliability of the results. However, not all mice are littermates, so the in vivo results need to be confirmed by further in vitro experiments because microbiota and genetic background have been shown to profoundly affect NLRP3‐mediated responses. The existing experimental results can help us make preliminary judgments that CD73 may play a crucial role in the regulation of pyroptosis driven by NLRP3 inflammasome after SCI and further experiments need to be conducted.

Microglia are vital mediators of the innate immune response following CNS trauma and are essential for subsequent inflammatory responses.^36^, ^37^ There exists a substantial amount of evidence supporting that pyroptosis mainly occurs in microglia in CNS neuroimmune diseases. A possible reason is that these cells express higher levels of PRRs which can recognize PAMPs and DAMPs, and initiate pyroptosis cascade.^10^, ^18^ We also observed intense GSDMD immunostaining in CD68‐labeled microglia and a profound increase in spinal cord lesions of CD73 KO mice (Figure 2G). Taken together, the present results defined a pyroptosis occurring in microglia could be inhibited by CD73 in SCI.

CD73, a glycosylphosphatidylinositol anchored cell surface protein, has a central role in the adenosinergic system and is considered as the rate‐limiting enzyme in the generation if extracellular adenosine.^38^ Kulesskaya et al found about 85‐95% of murine AMP‐hydrolyzing capabilities are mediated by CD73.^39^ When the concentration of extracellular adenosine increases, it can activate P1 purinergic receptors (adenosine receptors) on target cells and stimulates a myriad of protective cellular responses restoring homeostasis.^40^, ^41^ The P1 G‐protein‐coupled receptor family consists of four distinct subtypes: A_1_R_,_ A_2A_R, A_2B_R, and A_3_R.^42^ However, these four receptors have different affinities with adenosine. A_1_R_,_ A_2A_R, and A_3_R are categorized as high‐affinity adenosine receptors because they can be activated by physiologic adenosine concentrations, while A_2B_R can only be activated under pathological conditions, which is relevant to inflammatory events.^43^, ^44^ Experimental evidence reveals that hypoxia can increase the expression of A_2B_R,^45^ resulting in the activation of A_2B_R signaling to protect rats lung injury^46^ as well as myocardial ischemia.^47^ Similarly, we confirmed CD73 attenuate SCI in mice by A_2B_R in our previous research.^24^ Taken together, we assumed that adenosine‐A_2B_R cascade may be one of the mechanisms by which CD73 regulates the pyroptosis of microglia after SCI. In the subsequent study, our data showed that the effect of CD73 interference or overexpression in BV2 cells can be counteracted by adenosine or MRS1706, and these results are consistent with our hypothesis (Figure 3).

To further investigate the mechanism by which CD73 regulates microglia pyroptosis, mRAN sequencing was performed on CD73‐overexpressing BV2 cells after administrated with LPS. KEGG analysis showed a large number of differential genes enriched in the PI3K/AKT pathway. PI3K/AKT signaling pathway plays a central role in multiple cellular functions such as cell proliferation and survival.^48^ More noteworthy is that the activation of this pathway is closely related to the inflammatory response. For example, the research of Yin et al demonstrated the expression of pro‐inflammatory factors IL⁃12, TNF⁃α, and IL⁃6 in human innate immune cells was increased by PI3K or AKT inhibitors, while the expression of anti‐inflammatory factor IL⁃10 was decreased.^49^ Another study showed that the activation of AKT inhibited inflammatory responses in LPS‐induced sepsis mice and sepsis rabbits.^50^ These studies reflect the immunosuppressive function of the PI3K/AKT pathway, which is consistent with the role of CD73. To explore the downstream of PI3K/AKT, we detected the common downstream signaling molecules of PI3k/AKT signal axis (including mTOR, IKK‐β, GS3K‐β, Bad, and Foxo1) in CD73 overexpressed BV2 cells (Figures 4D and 4E). The results showed that only Foxo1 phosphorylation status can be affected by CD73. Moreover, Kayagaki et al reported that IRF2, a transcription factor, could induce GSDMD expression for pyroptosis at the transcriptional level.^51^ Therefore, we speculated that if the change of expression level of GSDMD is regulated by some transcription factors. The JASPAR database (http://jaspar.genereg.net) was utilized to predict the potential transcription factors of GSDMD. Interestingly, among these TFs, Foxo1 is one of the most common downstream signaling molecule of PI3k/AKT pathway in many previously published researches. Foxo1 is a vital transcription factor downstream of AKT. When PI3K/AKT pathway is constitutively activated, Foxo1 can be phosphorylated by AKT in the nucleus, resulting in the translocation of Foxo1 to the cytoplasm and inactivate it.^52^ A large number of studies have found that inhibition of Foxo1 can reduce inflammation in different cells.^53^, ^54^ To date, however, there are no reports about Foxo1 is related to CD73 or pyroptosis. In the present study, our results indicated that overexpression of CD73 alleviates the pyroptosis of microglia in a PI3K/AKT dependent way both in vivo and in vitro (Figure 4). Furthermore, the results of dual‐luciferase reporter assay in BV2 cells demonstrated that there is a Foxo1 binding site between 300 bp upstream of the GSDMD gene and 50 bp downstream, and this discovery was also verified by ChIP assay (Figure 5). Based on the above findings, we believe that the PI3K/AKT/Foxo1 pathway is essential for CD73 to regulate pyroptosis.

The fact that CD73 was overexpressed after SCI has been confirmed repeatedly in the present study as well as our previous research. However, the biological mechanism behind this is still unclear. HIF‐1 is a heterodimeric transcription factor composed of a HIF‐1α subunit and HIF‐1β subunit and functioned as a master regulator of oxygen homeostasis.^55^ When in anoxic conditions, the HIF‐1α subunit accumulates and then binds to HIF‐1β, ultimately activates HIF‐1‐target genes, whose products are regulating angiogenesis, glucose metabolism, cell survival, invasion, and metastasis.^56^ Numerous studies suggested that the pathogenesis of SCI involves the ischemia and hypoxia in lesion site, which increases the expression of HIF‐1, enhancing the resilience of neuronal cells under hypoxia.^57^, ^58^ Karhausen et al once reported activation of HIF‐1α promoted CD73 transcription, resulting in attenuated loss of barrier during colitis in vivo.^59^ Besides, Synnestvedt et al examined the CD73 gene promoter and identified a binding site for HIF‐1α, and inhibition of HIF‐1α expression by antisense oligonucleotides resulted in significant decrease of hypoxia‐inducible CD73 expression.^27^ In this study, we also found that overexpression of CD73 after SCI is associated with HIF‐1α. Interfering with the expression of HIF‐1α or inhibiting the activity of HIF‐1α can reduce the expression of CD73 after SCI (Figures 6C and 6J). Interestingly, we also found that the expression of HIF‐1α in microglia was CD73 dependent. The adenosine‐A_2B_AR‐p38 pathway, which has been confirmed, regulated the function of macrophages/microglia after SCI in our previous study, probably the mechanism by which CD73 regulates HIF‐1α expression (Figures 6K and 6S). This finding is consistent with that reported by Merighi.^28^ Therefore, we believe that a positive feedback regulatory loop can be formed between CD73 and HIF‐1α after SCI, which may be involved in the regulation of microglia pyroptosis and inhibits neuroinflammatory response.

A limitation can't be neglected in this study is the alternative of BV2 cell line as primary microglia. BV2 cells were derived from raf/myc‐immortalised murine neonatal microglia and are the most frequently used substitute for primary microglia. Although there exits research confirmed BV2 cells appear to be a valid substitute for primary microglia in many experimental settings including complex cell‐cell interaction studies,^60^ however, doubts have been raised that this cell line does not always model the reaction of primary microglia.^60^, ^61^ Another limitation is that we didn't confirm the correlation between NLRP3 inflammasome and pyroptosis in microglia. In addition to NLRP3 inflammasome, the activation of Caspase‐11 can also cleavage GSDMD and triggers pyroptosis in rodents.^62^, ^63^ Although the gene level of Caspase 11 didn't show significant difference between SCI and sham group, its role in pyroptosis after SCI is still worthy of further study. Therefore, although the present study is helpful in understanding the mechanism of microglial pyroptosis, further experimental verification is still needed.

## 5 CONCLUSION

**FIGURE 7:**
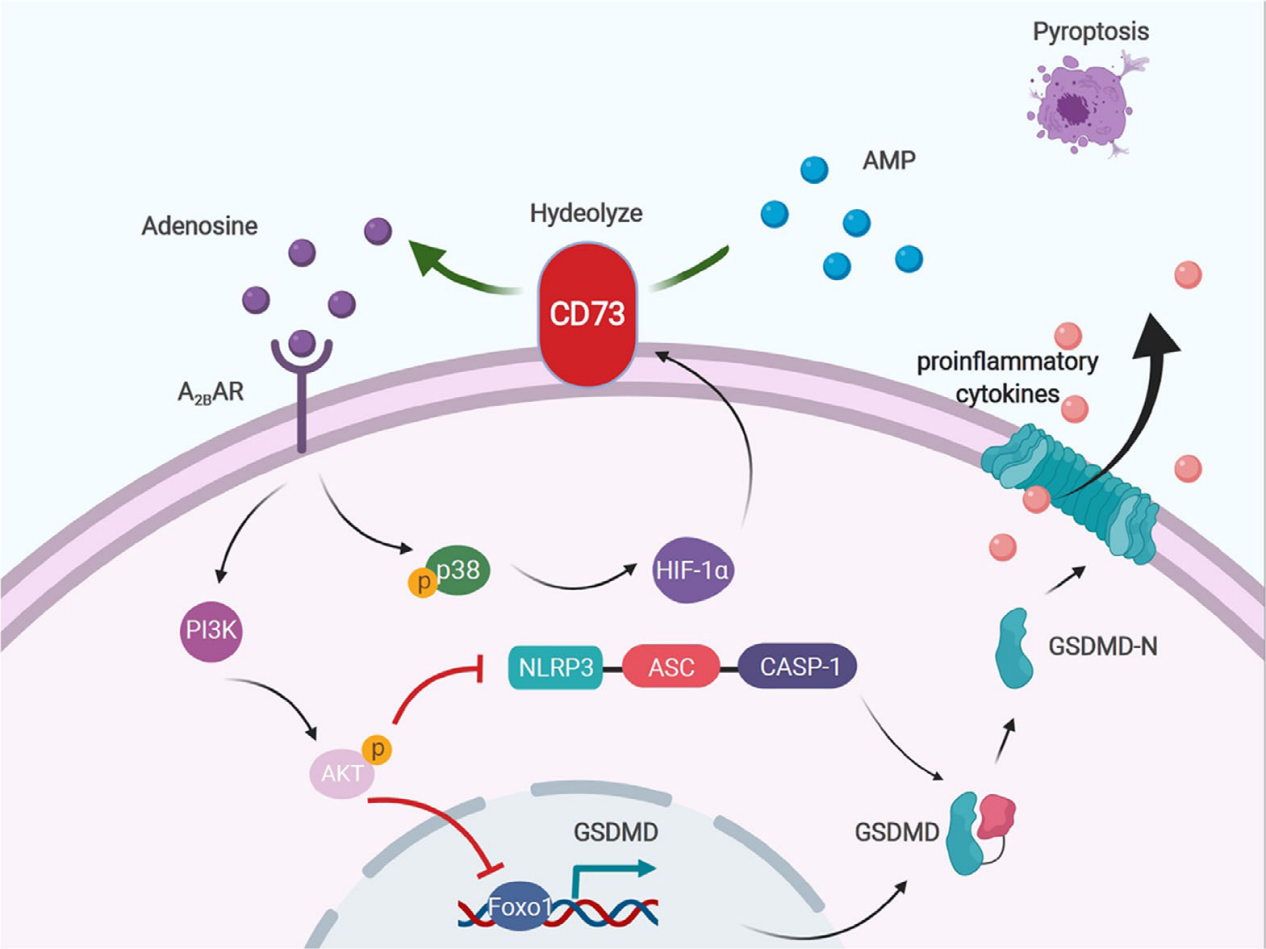
CD73 alleviates GSDMD‐mediated pyroptosis through inhibiting PI3K/AKT/Foxo1 signaling. CD73 promotes an increase in the concentration of extracellular adenosine after injury, increases PI3K/AKT activation through the A_2B_ adenosine receptor, thereby blunting NLRP3 inflammasome activation and reducing GSDMD transcription. The accumulation of HIF‐1α after spinal cord injury facilitates the upregulation of CD73, while the overexpressed CD73 promotes the further aggregation of HIF‐1α through adenosine‐A_2B_AR‐p38 cascade, forming a positive feedback regulation. This picture is drawn with BioRender (www.biorender.com)

In summary, our present study demonstrates for the first time that CD73 has an anti‐pyroptosis role in SCI, which is partly attributable to its inhibition of GSDMD via adenosine‐ A_2B_AR‐PI3K‐AKT‐Foxo1 signal. Moreover, our results showed that the creation of a positive feedback loop between CD73 and HIF‐1α may an important mechanism for reducing neuroinflammation after SCI (Figure 7). We propose that CD73 may be utilized as a target molecule for the development of novel therapeutic methods for SCI.

## CONFLICT OF INTEREST

We declare that we do not have any commercial or associative interest that represents a conflict of interest in connection with the work submitted.

## ETHICS APPROVAL AND CONSENT TO PARTICIPATE

All study surgical procedures and experiment protocols have been approved by the Ethics Committee of Experimental Research, Shanghai Medical College, Fudan University (number: 2019‐JS‐053). All participants have been informed the potential risks and benefits of the study, and each patient has signed an informed consent form before blood sample collection.

## Supporting information

Supporting Information

Supporting Information

## Data Availability

The data that support the findings of this study are available from the corresponding author upon reasonable request.
