# Supplementary figures and images for: CD73 alleviates GSDMD‐mediated microglia pyroptosis in spinal cord injury through PI3K/AKT/Foxo1 signaling
Source: Clin Transl Med. 2020 Dec 31;11(1):e269. doi: 10.1002/ctm2.269 (PMC7774461; doi:10.1002/ctm2.269)

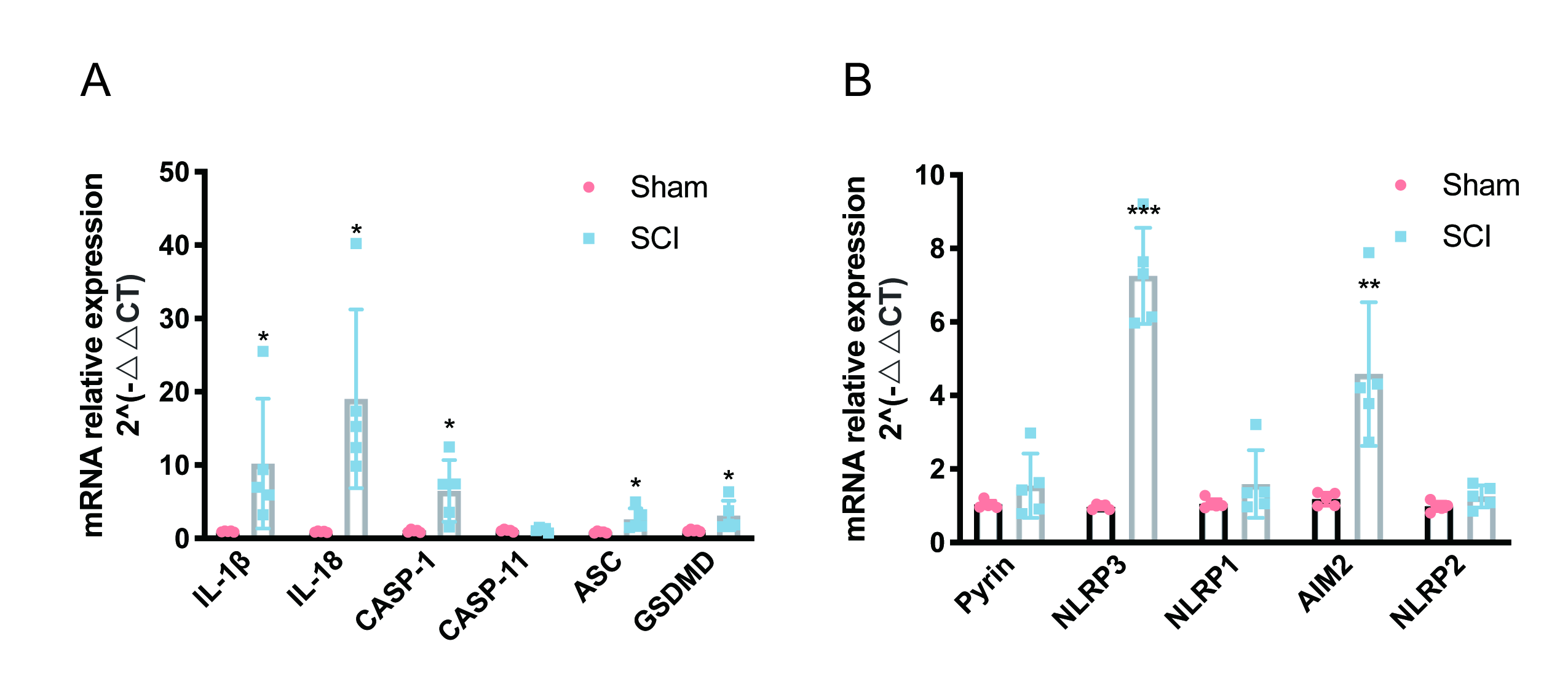

Supplement: Supplementary file 1 — Supporting Information [file CTM2-11-e269-s001.tif]
